# Acute Large Bowel Obstruction Caused by Endometriosis Requiring Sigmoidectomy

**DOI:** 10.7759/cureus.32430

**Published:** 2022-12-12

**Authors:** Patrick D Plummer, Raydiene Doorgen, Benjamin Yglesias, Joshua K Phillips

**Affiliations:** 1 Department of Surgery, Trumbull Regional Medical Center, Warren, USA

**Keywords:** open decompression, sigmoidectomy, sigmoid stricture, large bowel obstruction, laparoscopic surgery for endometriosis

## Abstract

Large bowel obstruction (LBO) accounts for nearly 25% of all bowel occlusions. LBO is managed as a surgical emergency due to its increased risk of bowel perforation. Nearly, 2% to 4% of all surgical admissions are a result of LBO. The most common pathological development of LBO remains colonic malignancy, representing approximately 60% of cases. Other etiology includes abdominal adhesions, diverticulosis, hernia, inflammatory bowel disease (IBD), and in rare cases endometriosis. In this report, the patient is a 36-year-old female with an LBO, originally thought to be a complication of diverticulitis. However, it was confirmed that the obstruction was a result of endometriosis tissue adherence to the colonic wall narrowing the intestinal lumen. The patient presented to the emergency department (ED) with nausea, vomiting, and abdominal pain that started six weeks prior. In this case report, we will discuss the rare complication of endometriosis causing LBO, clinical presentation, diagnosis, and management.

## Introduction

Large bowel obstruction (LBO) is classified into three distinct categories: partial or complete, intrinsic or extrinsic, and benign or malignant [1]. Nearly, 75% of LBOs are located distally to the transverse colon where there is a decrease in luminal size [2]. While LBOs are less common than small bowel obstructions (SBOs), LBO presents with an increased risk of perforation and diffuse peritonitis [1,3,4].

In the United States, adenocarcinoma of the colon and rectum is the most common cause of bowel obstruction representing 50% to 60% of all cases [1]. The common location for colorectal cancer is the sigmoid colon. Tumors at the splenic flexure are associated with a high prevalence of obstruction compared with hepatic flexure tumors [2]. In addition, 10% to 20% of LBO cases are due to diverticular diseases [1]. Volvulus contributes 10% to 15% of the causes of the development of LBOs. A small percentage of factors causing LBOs can be inflammatory bowel disease (IBD), hernia, adhesion, endometriosis, and intussusception [1,3].

Intrinsic LBOs are associated with clinical conditions such as Crohn’s disease and ulcerative colitis. The inflammatory changes of the mucosa, submucosa, or serosa cause thickening of the bowel, leading to strictures [5]. The acute inflammation of diverticulitis over time leads to luminal scarring and fibrosis, which gradually narrows the lumen.

The most common etiology of extrinsic LBO in industrialized nations is postsurgical adhesions. It is estimated that two-thirds of patients with a past medical history of abdominal surgery will have symptomatic or asymptomatic adhesions. Large intestine adhesion causes torsion and strictures of the bowel, leading to obstruction. Intestinal herniation through the abdominal wall or umbilicus results in strangulation or incarceration of the bowel, leading to ischemia and obstruction of the large or small bowel [5].

The clinical presentation of acute LBOs usually presents abruptly or after one to two days of symptoms. The patient can present with bloating, abdominal pain, and constipation. The characteristic of abdominal pain is described as infraumbilical, with cramping occurring every 20 to 30 minutes [2]. The presence of nausea and vomiting is dependent upon the location of the obstruction. Usually, proximal colonic obstruction presents similarly to SBOs and is associated with nausea and vomiting [2]. Abdominal examination reveals distention with focal or diffuse tenderness. In the case of severe LBOs, the patient may show signs of dehydration or shock, resulting in tachycardia or hypotension [2]. 

The diagnostic modalities used to diagnose LBOs include plain radiography (X-ray) and CT scan [5]. In hemodynamically stable patients, abdomen CT has a high sensitivity and specificity for detecting LBO [5]. While X-ray is more cost-effective, it has a low sensitivity and specificity. The initial management of LBO begins with the resuscitation of isotonic intravenous fluids and the correction of metabolic abnormalities [5]. Antibiotics are administered to patients showing signs of sepsis, ischemia, or perforation. In patients with peritonitis or sepsis, emergency laparotomy or laparoscopy is considered the first-line treatment [5].

## Case presentation

The patient is a 36-year-old female with a past medical history of endometriosis, a brain tumor diagnosed at the age of 9 years and treated with radiation and chemotherapy, bipolar disorder, seizures, and a hysterectomy 10 years ago. The patient presented to the emergency department (ED) with diffuse abdominal pain, nausea, and vomiting that began six weeks prior and had progressively worsened. The patient had experienced nausea and vomiting after most meals, which consisted mainly of undigested food. She complained of diffuse abdominal pain centered in the lower abdomen with no radiations. The patient also denied passing of flatulence or having a bowel movement for one day. The patient reported a weight loss of 80 lbs over the past six to eight months. Physical examination revealed a rigid abdomen tender in all four quadrants, with no rebound or guarding. The patient's comprehensive metabolic panel (CMP) on arrival is given in Table 1. The patient's complete metabolic panel during admissions is given in Table 2. 

**Table 1:** The patient’s CMP taken during her hospitalization before surgery. ALT, alanine aminotransferase; CMP, comprehensive metabolic panel; AST, aspartate aminotransferase; BUN, blood urea nitrogen

CMP	Patient labs	Reference range
Sodium (mmol/L)	142	136-145
Potassium (mmol/L)	4	3.5-5.1
Chloride (mmol/L)	109	98-107
Carbon dioxide (mmol/L)	26	21-32
Anion gap (mmol/L)	11	10-22.1
BUN (mg/dL)	13	7-18.1
Creatinine (mg/dL)	0.83	0.50-1
Estimated creatinine clear (mL/min)	11	>60
Glucose (mg/dL)	95	70-99
Lactic acid (mg/dL)	1.3	0.5-2.2
Calcium (mg/dL)	8.6	8.5-10.1
Phosphorus (mg/dL)	3.6	2.5-4.9
Magnesium (mg/dL)	2.1	1.7-2.4
Total bilirubin (mg/dL)	0.5	0.2-1
AST (mg/dL)	78	10-37.1
ALT (unit/L)	127	12-78.1
Alkaline phosphatase (unit/L)	117	45-117
Total protein (gm/L)	7.3	6.4-8.2
Albumin (gm/L)	3.8	3.4-5
Globulin (gm/L)	3.5	2.5-5
Albumin/globulin ratio	1.1	1-2

 

**Table 2:** The patient’s CBC taken during her hospitalization before surgery. CBC, complete blood count; WBC, white blood cell; RBC, red blood cell; Hgb, hemoglobin; Hct, hematocrit; MCV, mean corpuscle volume

CBC	Patient lab	Reference range
WBC (fL)	7.9	81.5-98.3
RBC (pg)	4.25	26-34
Hgb (gm/dL)	13	12-15.5
Hct (%)	40	34.9-44.5
MCV (fL)	95.1	81.6-98.3
Neutrophils (%)	38.5	34-71
Lymphocytes (%)	46.1	15-44
Monocytes (%)	13.7	0-10
Eosinophils (%)	0.6	0-7
Basophils (%)	0.5	0-2

**Figure 1:**
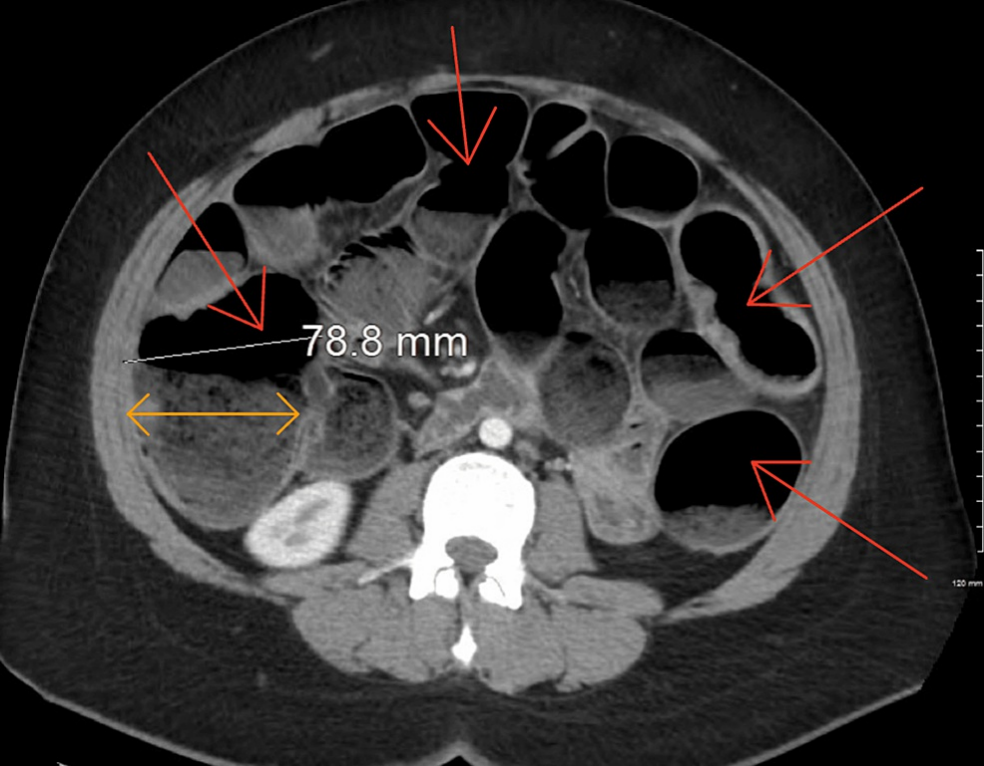
Axial CT scan. Red arrows show severe distention of the small and large bowels. The orange arrow shows distention of the colon, with the cecum measuring nearly 8 cm in diameter. CT, computed tomography

**Figure 2:**
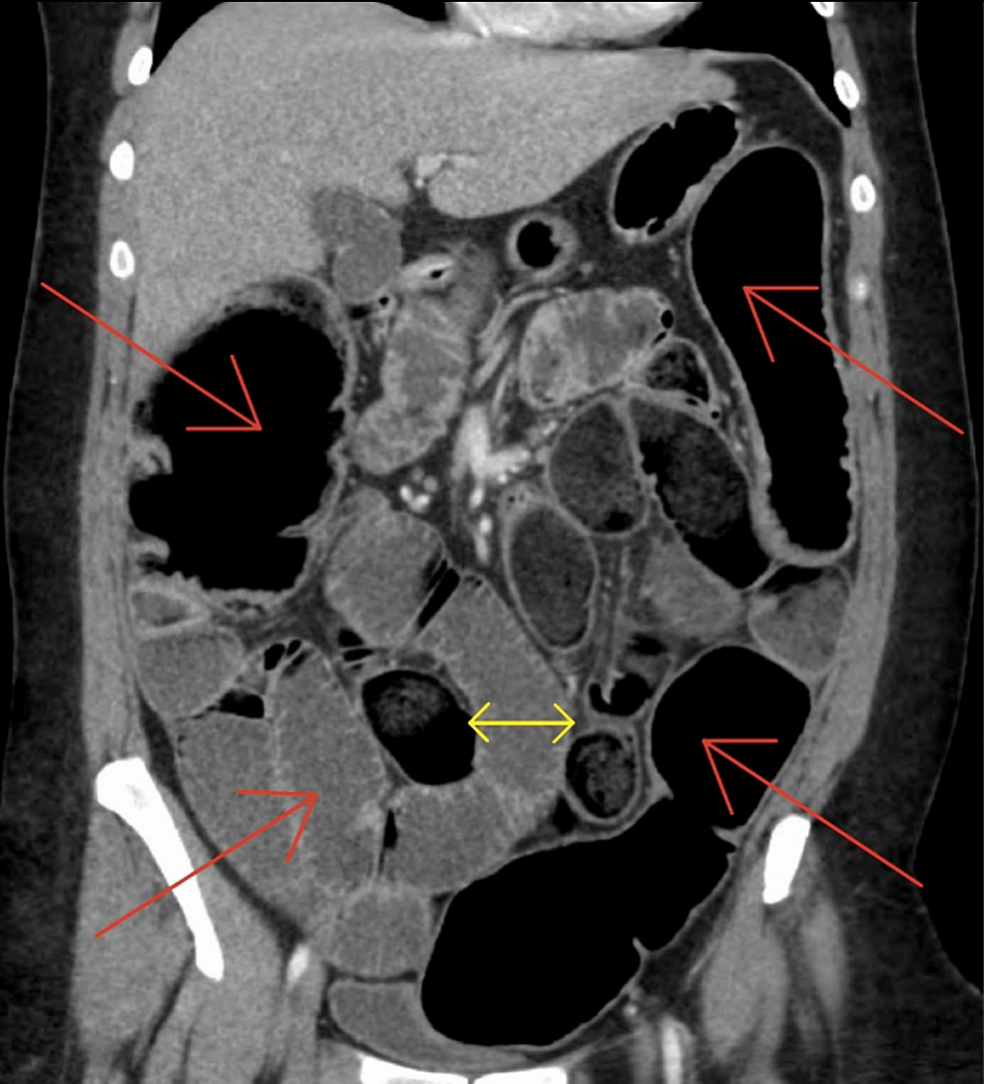
Coronal CT scan. Red arrows show severe distention of the small and large bowels. Yellow arrows show distention of the small bowel greater than 4 cm in diameter. CT, computed tomography

The results from the CT scan indicated diffuse small and large bowel dilatations (Figures 1, 2). The attempt to manage the patient's bowel obstruction through noninvasive procedures failed. The nasal gastric tube was unsuccessful in the efforts to help decompress the intestines. In consultation with the surgical team, the decision was made to perform a surgical intervention to avoid bowel ischemia or perforation. Given the amount of bowel dilatation, a laparotomy was performed. Upon entry into the abdominal cavity, visualization was poor, and the procedure was converted to an open laparotomy.

The small and large bowels were examined for ischemia or perforations. The sigmoid colon was located, and a distal sigmoid stricture was visualized. The proximal and distal attachments of the sigmoid colon were slowly dissected, with special emphasis on avoidance of injury to the ureter and retroperitoneum. During the dissection of the distal sigmoid, the right ovary and fallopian tube had significant adherence to the sigmoid wall. Dark brown ovarian cysts were encountered during the distal dissection. After complete mobilization, the sigmoid colon was resected and sent to pathology. The distal portion of the rectum was closed creating a rectal stump. Due to severe dilatation and the size difference between the descending colon and the rectum, we decided to create an end colostomy rather than perform a primary anastomosis. The descending colon was then used to create an end colostomy. The midline incision was closed, and the patient was extubated and transferred to the recovery room for further monitoring. The patient had a stable postoperative course. The end colostomy was functional on postoperative day 2, and her diet was advanced as tolerated. She was discharged in stable condition with no immediate complications.

**Figure 3:**
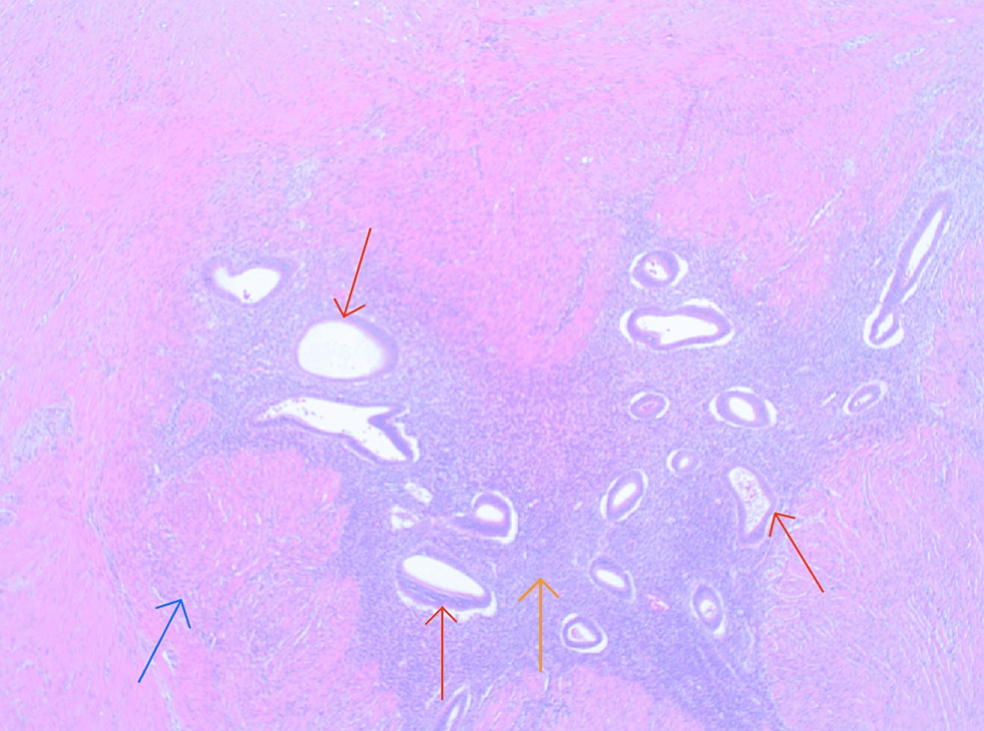
Red arrows show endometrial tissue with a glandular structure embedded in the muscular layer of the colonic wall. The orange arrow shows the surrounding stroma. The blue arrow shows the muscular layer of the colon.

The pathology findings of the resected sigmoid colon showed endometriosis and endometrioma with endometriotic cysts and serosal adhesions involving the muscular layer of the colon. At the site of the sigmoid stricture, revealed a firm 3 cm × 3 cm × 3 cm mass below the mucosa within the muscular wall. Histology of the colonic mass showed glandular endometriotic cysts with central stroma (Figure 3).

## Discussion

Endometriosis is found in 6% to 10% of women of reproductive age [6]. Nearly 50% of women with this disorder experience pelvic pain, abdominal menstruation, and infertility [6]. The common location of external endometrial tissue includes the pelvic peritoneum, ovaries, and rectovaginal septum [6]. In rare cases, the pleura, pericardium, small and large intestines, and diaphragm can become involved. In 12% of women with endometriosis, the most common extrapelvic site is the intestines [6]. The rectosigmoid junction accounts for 72% of intestinal endometriosis cases [6]. Endometriosis tissue causing LBOs remains a rare condition, with an incidence between 0.1% and 0.7% [6].

Endometriosis is a benign gynecological disease caused by endometrial tissue located outside of the uterus [7]. The endometrial tissue can be located throughout the peritoneal cavity, especially in the rectosigmoid junction [6]. According to Rolla [8] and Sampson [9], the pathogenesis of endometriosis is not fully understood but is theorized to be contributed to the retrograde flow of menstrual blood mixed with endometrial tissue through the fallopian tube into the peritoneal cavity. This condition is sought to develop in female infants due to a drastic decrease in hormonal estrogen and progesterone after birth, triggering the shedding of the endometrial tissue [10]. The presence of a tight cervical junction, thick cervical mucus, or malformations of the female reproductive tract prevents the expulsion of endometrial tissue, leading to retrograde flow [10]. The endometrial tissue inside the peritoneum remains dormant until the onset of puberty when the tissue is reactivated by surging hormonal levels.

The diagnosis of intestinal endometriosis can be difficult due to the overlapping of nonspecific symptoms. The onset of lower abdominal pain and change in bowel habits can resemble adenocarcinoma of the colon, which is the leading cause of LBO. Diagnostic modalities such as the abdomen and pelvic CT scans can reveal thickened colonic walls and impending strictures [6]. While the preferred modality for diagnosis of endometriosis includes transvaginal ultrasonography (US) and pelvic magnetic resonance imaging (MRI), they perform poorly at identifying specific tissue deposits and colonic adhesions [6]. Typically, endoscopic evaluation of the intestines for adherence of endometrial tissue shows no abnormality. The location of the endometrial tissue is in the serosa and muscularis layers of the colon, sparing the mucosa [6]. The gold standard diagnostic tool used to identify endometrial tissue adherence to the colonic wall remains laparoscopy with biopsy [6]. This procedure was seen in the patient mentioned in this report, which allows a full assessment of the pelvis and tactical surgical advantage if required.

In this case report, the main question that is presented in patients with endometriosis-induced LBO is if effective medical management of their endometriosis could have prevented the formation of the obstruction. The medical management of patients with endometriosis is centered around controlling the patients' dysmenorrhea. First-line therapy includes nonsteroidal anti-inflammatory drugs (NSAIDs) in combination with oral contraceptive pills (OCPs) [6]. The addition of OCPs help control the cyclic growth and shedding of the endometrial tissue in the response to fluctuating levels of estrogen and progesterone during the ovarian cycle. OCPs have been proven to help decrease the symptoms and size of ovarian endometriomas [11]. Other management therapies include gonadotrophin-releasing hormone (GnRH) agonists and aromatase inhibitors [12]. These medications help depress the ovarian-hypothalamus-pituitary axis, resulting in a hypoestrogenic state and endometrial atrophy [12].

## Conclusions

Endometriosis-induced LBO is a rare complication seen in women of reproductive years. It remains a difficult condition to diagnose due to the physiological adhesion of the endometrial tissue to the colonic wall. While diagnostic modalities such as US and MRI are effective at evaluating extrapelvic endometrial tissue, the extent of the invasion cannot be visualized with imagining studies. In female patients, with a past medical history of endometriosis who are showing signs and symptoms of bowel obstruction, endometriosis-induced bowel obstruction should be a top differential. In patients with endometriosis, it is of high importance to provide effective pharmacological management to help control pain and reduce the size of the endometrial tissue. Early diagnosis and management of endometriosis can help decrease the risk of related complications, including LBO.
